# A Single Neonatal Exposure to BMAA in a Rat Model Produces Neuropathology Consistent with Neurodegenerative Diseases

**DOI:** 10.3390/toxins10010022

**Published:** 2017-12-29

**Authors:** Laura Louise Scott, Timothy Grant Downing

**Affiliations:** Department of Biochemistry and Microbiology, Nelson Mandela University, P.O. Box 77 000, Port Elizabeth 6031, South Africa

**Keywords:** β-*N*-methylamino-l-alanine, BMAA, rats, neurodegeneration, Alzheimer’s disease, amyloid, neurofibrillary tangles, Lewy bodies, Parkinson’s disease

## Abstract

Although cyanobacterial β-*N*-methylamino-l-alanine (BMAA) has been implicated in the development of Alzheimer’s Disease (AD), Parkinson’s Disease (PD) and Amyotrophic Lateral Sclerosis (ALS), no BMAA animal model has reproduced all the neuropathology typically associated with these neurodegenerative diseases. We present here a neonatal BMAA model that causes β-amyloid deposition, neurofibrillary tangles of hyper-phosphorylated tau, TDP-43 inclusions, Lewy bodies, microbleeds and microgliosis as well as severe neuronal loss in the hippocampus, striatum, substantia nigra *pars compacta*, and ventral horn of the spinal cord in rats following a single BMAA exposure. We also report here that BMAA exposure on particularly PND3, but also PND4 and 5, the critical period of neurogenesis in the rodent brain, is substantially more toxic than exposure to BMAA on G14, PND6, 7 and 10 which suggests that BMAA could potentially interfere with neonatal neurogenesis in rats. The observed selective toxicity of BMAA during neurogenesis and, in particular, the observed pattern of neuronal loss observed in BMAA-exposed rats suggest that BMAA elicits its effect by altering dopamine and/or serotonin signaling in rats.

## 1. Introduction

Guamanian Amyotrophic Lateral Sclerosis/Parkinsonism-Dementia Complex (ALS/PDC) was first described based on clinical symptoms and neuropathology that resemble aspects of Parkinson’s Disease (PD), Alzheimer’s Disease (AD) and Amytrophic Lateral Sclerosis (ALS) [1,2,3,4]. The neuropathology is characterised by abundant neurofibrillary tangle (NFT) pathology, neuronal loss, amyloid plaques that resemble those observed in AD patients [2,5,6] together with alpha-synuclein pathology in the substantia nigra and cerebellum [7] and reactive glial cells and pathological TDP-43 positive inclusions in hippocampal neurons and motor neurons [8].

Dietary exposure to the cyanobacterial toxin, β-*N*-methylamino-l-alanine (BMAA) has been suggested to be involved in the aetiology of Guamanian ALS/PDC [9,10,11], AD, PD and Amyotrophic Lateral Sclerosis (ALS) [12]. This proposed BMAA-ALS/PDC link is based largely on the presence of BMAA in traditional dietary items of the Chamorro people of Guam [12,13,14] and on the reported presence of BMAA in the brain tissue of Chamorro ALS/PDC, Canadian AD, sporadic ALS and PD patients [15,16,17].

Although some clinical signs of BMAA toxicity have been shown in chicks, rats and mice [9,18,19,20,21,22,23] with symptoms such as convulsions and ataxia being reported at high doses (4000 mg per kilogram body weight) [21], no animal model of BMAA toxicity has successfully reproduced all the neuropathological changes that are typically seen in AD, PD and/or ALS patients. Neither Polsky et al. [24], nor Perry et al. [25], nor Duncan et al. [26] nor Cruz-Aguado et al. [27] observed any clinical signs of toxicity, or biochemical or neuropathological abnormalities associated with the administration of BMAA to adult rats. Seawright et al. [21] were able to show some degeneration of cerebellar stellate, basket, and Purkinje cells in adult rats following injection with 1000 mg/kg BMAA. These findings were corroborated by Staton and Bristow [28] and later by Munoz-Saez et al. [19] who demonstrated swelling, vacuolization, mitochondrial and ER fragmentation and dislocation of the Golgi apparatus of the cerebellar Purkinje cells of adult rats treated with 300 mg/kg l-BMAA daily for five consecutive days. These are consistent with what is usually observed in motor neurons of the brain stem and spinal cord of patients with neurodegeneration [29], and dislocation of the Golgi apparatus of the cerebellar Purkinje cells is frequently reported to be present in patients with AD or ALS [30]. Lindstrom et al. [31], while unable to demonstrate clinical signs of neurological abnormalities, observed abundant gliosis and pyknotic neurons surrounding the BMAA injection site as well as a dose-dependent loss of tyrosine hydroxylase (TH)-immunoreactive neurons and dendrites in the lesioned areas one week following intranigral or intracisternal injections of 10 μg or 400 μg BMAA to adult rats. However, although Al-Sammak et al. [18] observed clinical symptoms in adult mice, such as myoclonus, convulsions, dragging gait, shaking and ataxia, following exposure to 2000 mg/kg body weight BMAA, and subsequently detected BMAA in the brain and liver tissue of all treated animals, histopathological examination of relevant brain tissue showed no histopathologic lesions in the brain. The livers, hearts, kidneys, lungs, and spleen tissue of BMAA-exposed animals were similarly unaffected. Since the brain tissue of symptomatic mice had no histopathological deficits, Al-Sammak et al. [18] suggested that BMAA might elicit its effect by causing biochemical changes rather than causing histopathologic lesions in the brain, although how this might occur is not clear. Additionally, no neuropathology was observed in rats injected subcutaneously with 200 mg/kg body weight BMAA on PND10 and mild neuronal loss could be seen only in the hippocampus of rats exposed to 600 mg/kg body weight BMAA on PND9 and 10 [22,23,32]. Buenz and Howe [33] have likewise demonstrated that direct administration of BMAA to the mouse’s brain leads to frank neuronal loss only in the hippocampus, and subsequently proposed that BMAA could be selectively toxic to this neuronal population. In addition to hippocampal neuronal loss observed at six months, in four of seven rats exposed to 600 mg/kg BMAA on PND9 and 10 Karlsson et al. [32] also observed reactive microglial cells and intraneuronal calcium deposition in the CA1 region of the hippocampus, as typically observed in the brain following excessive activation of excitatory receptors [34]. However, Karlsson et al. [32] observed no histopathological changes in the striatum, substantia nigra, dentate gyrus, cingulate cortex or retrosplenial granular cortex, and staining with Congo Red for identification of amyloid and immunohistochemical staining for phosphorylated tau, both pathological hallmarks of AD, was reported to be negative. 

Yin et al. [35] demonstrated that intrathecal BMAA administration to adult rats induced selective degenerative changes in ventral horn motor neurons, with little dorsal horn pathology, as well as marked ventral horn astrogliosis and mild accumulation of pathological TDP-43 in the cytosol of some injured and degenerating motor neurons. This is thus the only existing study that has confirmed that BMAA causes neuropathology consistent with ALS in an animal model. However, this has not been replicated using a systemic route of administration. 

Experimental evidence of the full spectrum of neuropathology characteristic of ALS/PDC in a rodent model has therefore not been reported. The presence of some neuropathology following systemic BMAA administration to neonatal rats, but the very limited, or, more commonly, complete absence of abnormality following similar BMAA exposure to adult animals, suggests that BMAA is, at least in rodents, more toxic to the developing brain. 

Cox et al. [36] recently reported hyper-phosphorylated tau deposits and NFT formation in the entorhinal cortex, amygdala, motor cortex, frontal cortex, temporopolar cortex and occipital cortex, and sparse immunopositive β-amyloid deposits in the frontal, temporal and motor cortices following oral l-BMAA exposure of 210 mg/kg/day for 140 days to adult Caribbean-derived vervets (*Chlorocebus aethiops*). Interestingly, the Caribbean-derived vervets carry the ApoE gene which has an amino acid sequence corresponding to human Apo E4, the allele which significantly increases the risk of developing AD, with the consequence that these monkeys naturally develop extracellular β-amyloid plaque build-up with age [37,38]. No mention is made of any observed neuronal loss and/or degeneration, astrogliosis or Lewy body formation in the BMAA-exposed vervets [36]. Additionally, no observed BMAA-induced behavioural deficits or cognitive impairments were reported in this study. Interestingly, no NFT formation or β-amyloid deposits could be observed in the hippocampus or dentate gyrus of exposed vervets. The hippocampus is the major target of the AD hallmarks; NFT, β-amyloid and neuronal loss [39]. For the majority of AD patients, hippocampal atrophy and protein aggregates in this area are the earliest detectable symptoms of ongoing neurodegeneration and these have been incorporated in the diagnostic criteria for AD [40,41]. Thus, although the study conducted by Cox et al. [36] was the first to successfully show that BMAA exposure causes the formation of NFT and β-amyloid deposits in some brain regions of the Caribbean-derived vervets, several important characteristic ALS/PDC, AD and PD-like symptoms and neuropathology are lacking in the model.

Widely used animal models of AD and PD, such as the rotenone, paraquat and MPTP models, have thus far failed to match all of the deficits that are commonly seen in patients with BMAA-associated neurodegenerative diseases (reviewed in [42]). An ideal model of PD-related synucleinopathies would exhibit the cardinal signs of idiopathic PD: postural impairment, resting tremor, gait disorder, rigidity, a relatively selective loss of DA neurons of the substantia nigra *pars compacta*, a good response to DA replacement early in the disease process, and the presence of Lewy bodies [43,44]. Without a reliable, consistent and accurate animal model for ALS/PDC as well as AD, PD and ALS it is impossible to effectively study disease mechanism and mode or to conduct preclinical screening of therapeutics for these diseases.

With the lack of an animal model that has successfully demonstrated the causative role of BMAA in the neurodegenerative diseases ALS/PDC, AD, PD and/or ALS, and with the observed difference in response to BMAA exposure in neonatal and adult rats, we sought to investigate and compare the BMAA-induced neuropathological changes in male and female rats following a single exposure to BMAA on gestational day (G)14, PND3, PND4, PND5, PND6, PND7 and PND10 or after accumulative dosing on G14, PND5 and PND10. These exposure times were selected to target specific developmental stages in the rodent brain in order to identify the brain regions and/or processes involved in BMAA toxicity. Although Buenz and Howe [33] and Karlsson et al. [22,23,32] reported that the hippocampus is particularly vulnerable to BMAA toxicity, no study has investigated the relative toxicity before, during, and after hippocampal development. Prenatal BMAA exposure (G14) was therefore intended to target the developing brain just before the onset of formation of the hippocampal regions (G15.5) and the dentate gyrus (G18) [45,46,47]. Cognitive decline in AD and PD are typically associated with impaired neurogenesis in the hippocampus, as reviewed by Mu and Gage [45]. Neurogenesis forms the basis for the normal structure and function of the adult brain and plays a critical role in the formation of hippocampal-dependent spatial learning and memory function later in life. Postnatal BMAA exposure regimes thus aimed to target hippocampal and dentate gyrus neurogenesis, which peaks at PND3, 4 and 5 and substantially decreases at the start of PND7. More than 80% of the hippocampal cells are produced from PND3–5 [46,47]. Additionally, at PND0–2, the first distinct patches of dopamine fibres are distributed throughout the striatum after which dopaminergic innervation increases markedly throughout the developing brain [47,48]. Dopamine and serotonin modulate several aspects of neuronal development, including cell proliferation, migration and differentiation, and furthermore contribute to the development of pathways needed for movement, cognition and reward [49,50,51,52,53,54]. Altered dopaminergic and serotonergic signalling during development can therefore produce long lasting changes that contribute to neurodegenerative disorders such as AD and PD.

## 2. Results

### 2.1. General Findings

Scott and Downing [55] showed that rats exposed to 400 mg/kg BMAA on PND3, 4 and 5 exhibited several AD and/or PD-related behavioural and cognitive deficits as well as gait and postural abnormalities. In the current study we conducted a histopathological evaluation of brain and spinal cord tissue of rats with behavioural and cognitive deficits as per Scott and Downing [55]. These rats received a single dose, *via* subcutaneously injection, of 400 mg/kg BMAA on G14, PND3, 4, 5, 6, 7 and 10.

### 2.2. Brain Pathology

**Figure 1:**
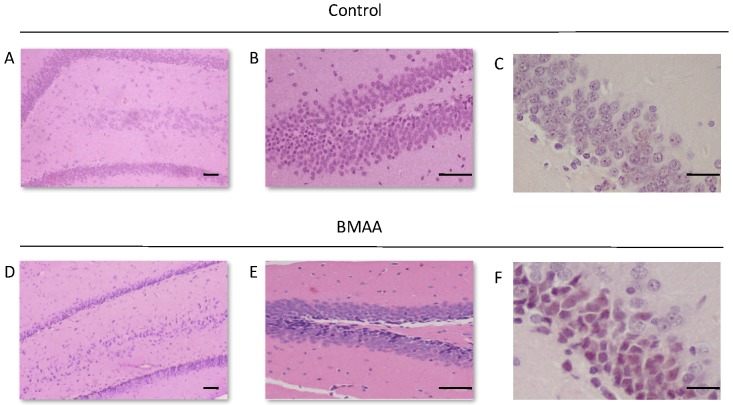
Dentate gyrus of a 120-day old male rat exposed to the vehicle control Hanks Balanced Salt Solution (HBSS) on PND3 (**A**), and the upper and lower blade of the dentate gyrus (**B**) and the dentate gyrus of a 120-day old female rat exposed to the vehicle control on PND5 (**C**) showing no observable neuronal loss and/or neuropathology, compared to the dentate gyrus of a 120-day old male rat exposed to 400 mg/kg β-*N*-methylamino-l-alanine (BMAA) on PND3 (**D**,**E**), and the dentate gyrus of a 120-day old female rat exposed to BMAA on PND5 (**F**). Note the thinning of the dentate gyrus blade in (**C**) compared to (**A**) as well as the darkly stained basophilic pyknotic and neurodegenerating neurons in the granular cell layer of the dentate gyrus of BMAA exposed rats in (**D**–**F**). Scale bars correspond to 50 μm for (**A**,**B**,**D**,**E**), and to 30 μm for (**E**,**F**). (**A**,**B**,**D**,**E**) are hematoxylin and eosin (H&E) stains, whereas (**C**,**F**) are cresyl violet Nissl stains.

**Figure 2:**
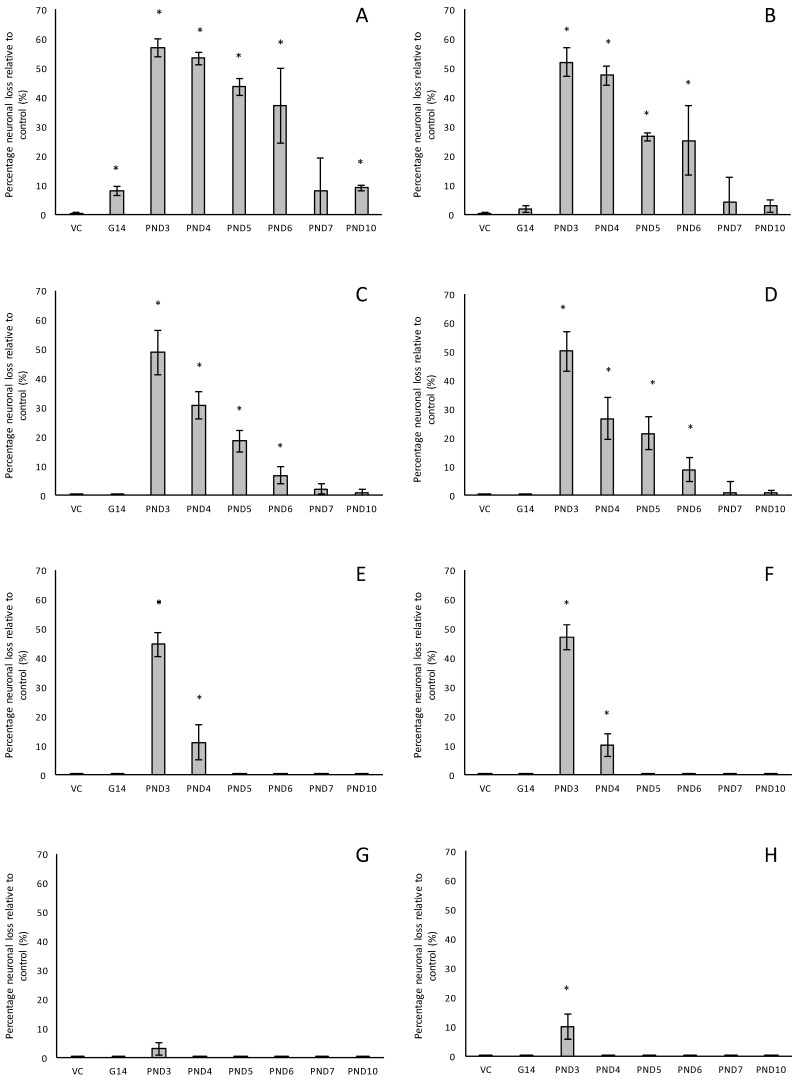
The percentage neuronal loss in 120-day old male (**A**,**C**,**E**,**G**) and female (**B**,**D**,**F**,**H**) Sprague Dawley rats following neonatal exposure to 400 mg/kg BMAA as observed in the hippocampus (**A**,**B**), striatum (**C**,**D**) and prefrontal cortex (**G**,**H**), and the percentage dopaminergic neuronal loss in the substantia nigra *pars compacta* in rats similarly exposed (**E**,**F**). Neurons in the hippocampus, striatum and prefrontal cortex were quantified using stereological counting of H&E and cresyl violet Nissl stained sections, and loss of dopaminergic neurons from the substantia nigra *pars compacta* visualised and quantified using immunostaining for tyrosine hydroxylase. For each exposure group *n* = 5, * indicates significant difference to the control (*p* < 0.05).

**Figure 3:**
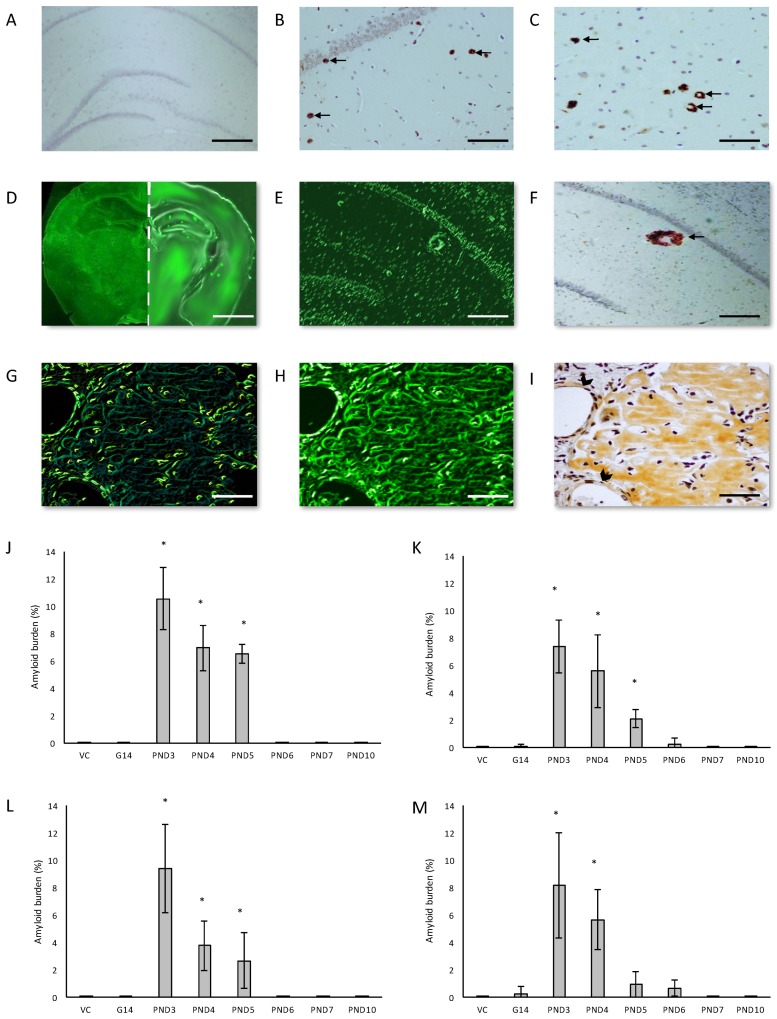
Anti-amyloid beta A4 antibody immunohistochemistry stains of B-amyloid plaque deposits in the hippocampus (**B**) and the prefrontal cortex (**C**) of a 120-day old male Sprague Dawley rat exposed to 400 mg/kg BMAA on PND3 compared to that of a 120-day old male rat exposed to vehicle control HBSS on PND3 (**A**). Thioflavin S-stained whole brain coronal section of a 120-day old male control rat (**D** (left panel)) and a 120 day old male rat exposed to BMAA on PND3 (**D** (right panel)) showing Thioflavin S-reactive areas in the BMAA-exposed rat and not in the control, which is magnified in (**E**) and confirmed to be a dense core amyloid plaque with immunostaining for beta amyloid using Beta A4 antibody (**F**). Serial sections of the striatum of a male Sprague Dawley rat exposed to BMAA on PND3 stained positive for beta amyloid deposition as shown by the apple green birefringence following a Congo Red stain (**G**) showing typical apple green birefringence of congophilic amyloid, Thioflavin S (**H**) and immunostained using beta A4 (**I**). Arrows indicate a few of the abundant B-amyloid deposits observed in the field of view. Note the b-amyloid deposition in cerebral blood vessels of the striatum in (**G**,**H**,**I**) which is indicated by an arrowhead in (**I**). Diffuse beta-amyloid deposition can also be observed in (**G**,**H**,**I**). The percentage amyloid burden, determined by stereological counting of immunostained sections as observed in the hippocampus (**J**,**K**), striatum (**L**,**M**) and prefrontal cortex (**O**) of male (*n* = 5) (**J**,**L**) and female (*n* = 5) (**K**,**M**,**O**) Sprague Dawley rats neonatally exposed to 400 mg/kg BMAA. * indicates significant difference to the control (*p* < 0.05). Scale bars correspond to 400 μm (**A**), 120 μm (**B**,**C**,**G**–**I**), 200 μm (**C**,**F**) and 3 mm (**D**).

**Figure 4:**
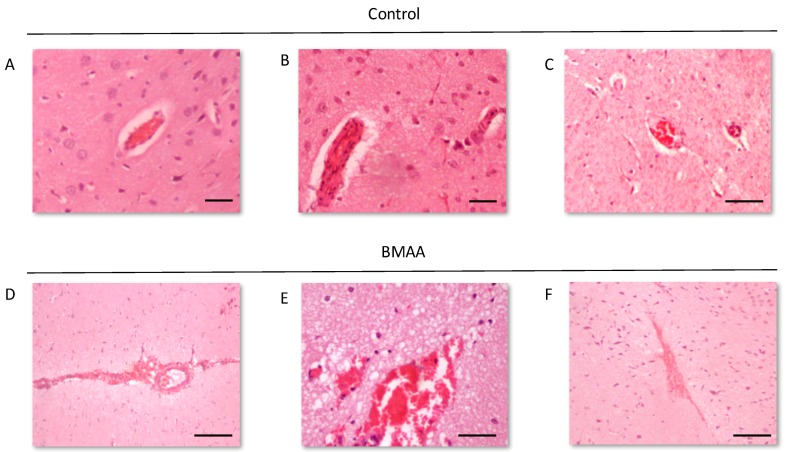
Intact blood vessels of rats exposed to vehicle control, HBSS, on PND3 compared to the ruptured blood vessels and microbleeds that can be observed in the striatum of male (**A**) and female rat (**B**,**C**) exposed to 400 mg/kg BMAA on PND3. Note the oedema surrounding the ruptured blood vessels. BMAA. Scale bars correspond to 20 μm (**A**,**B**) and 200 μm (**C**–**F**).

**Figure 5:**
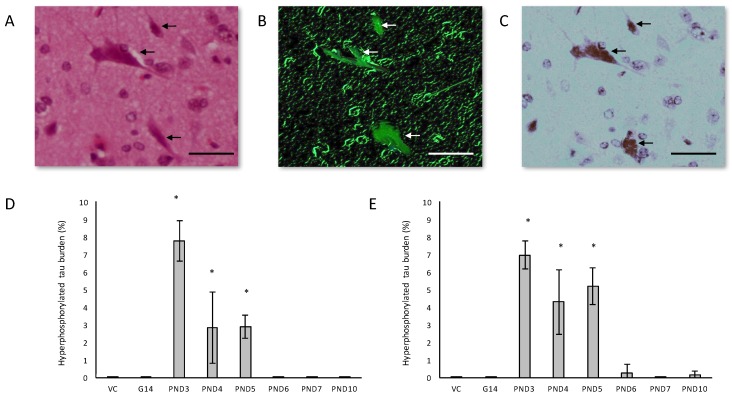
Healthy neurons are surrounded by neurons harboring classic flame-shaped neurofibrillary tangles (indicated by arrows) in serially stained sections of the hippocampus of a 120-day old female rat exposed to 400 mg/kg BMAA on PND5. Sections stained with H&E (**A**), Thioflavin-S (**B**) and immunostained using tau AT8 (**C**). Percentage hyperphosphorylated tau-positive burden, defined as the number of neurons in the hippocampus with tau-positive inclusions, for male (*n* = 5) (**D**) and female (*n* = 5) (**E**) rats. * indicates significant difference to the control (*p* < 0.05). Scale bars correspond to 20 μm.

**Figure 6:**
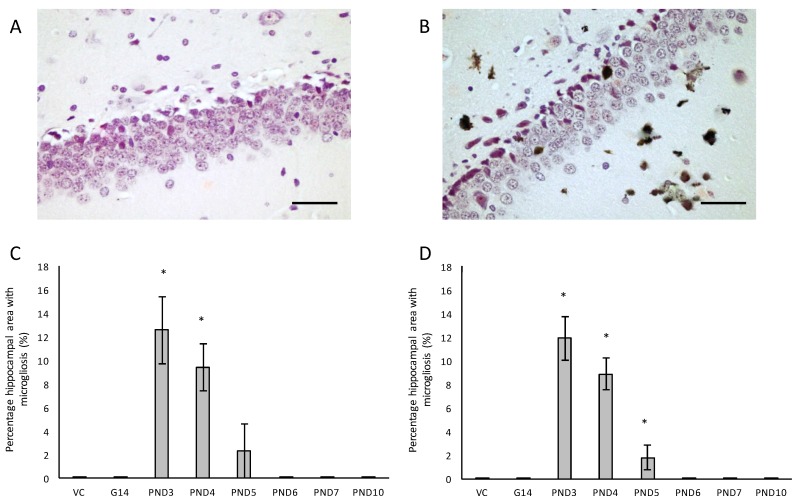
Immunostaining for reactive microglia in the hippocampus of a 120-day male rat exposed to vehicle control on PND3 (**A**) and that of a 120-day old male that was exposed to 400 mg/kg BMAA on PND3 (**B**). Microgliosis can be clearly seen throughout the parenchyma. The percentage hippocampal area with microgliosis is given for male (*n* = 5) (**C**) and female (*n* = 5) (**D**) rats. * indicates significant difference to the control (*p* < 0.05). Scale bars correspond to 30 μm.

**Figure 7:**
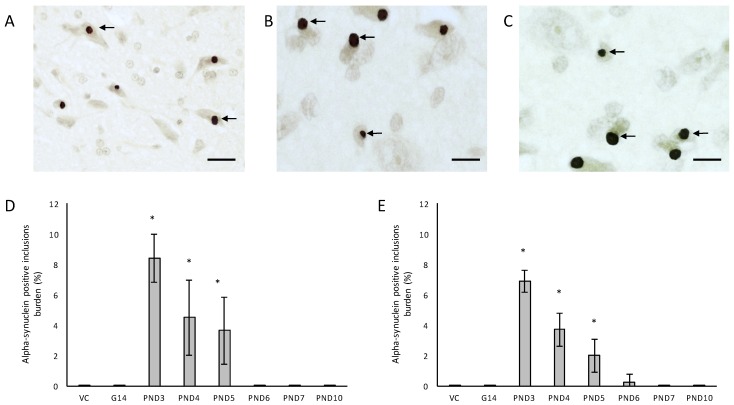
Immunostaining for α-synuclein: Lewy body inclusions (indicated by arrows) in the substantia nigra *pars compacta* of a 120-day old female rat exposed to BMAA on PND3 (**A**) and a 120-day old male rat exposed to 400 mg/kg BMAA on PND3 (**B**,**C**). The percentage alpha-synuclein positive burden, defined as the percentage neurons in the substantia nigra *pars compacta* that harbor Lewy body inclusions, is given for all male (*n* = 5) (**D**) and female (*n* = 5) (**E**) exposure groups. * indicates significant difference to the control (*p* < 0.05). Scale bars correspond to 25 μm (**A**) and 10 μm (**B**,**C**).

**Figure 8:**
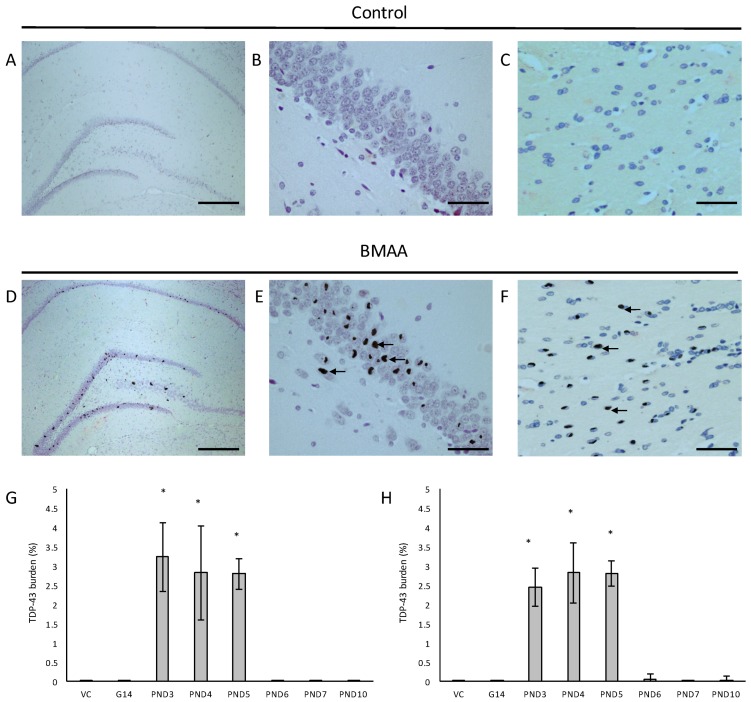
Neurons with pathological TDP-43 positive cytoplasmic inclusions (indicated by arrows) in the hippocampal formation (**D**–**F**) of male (**D**) and female (**E**,**F**) rats exposed to BMAA on PND3 and not in gender-matched rats exposed to the vehicle control on PND3. The percentage pathological TDP-43 burden, defined as the percentage neurons in the hippocampus that harbor hyperphosphorylated TDP-43 positive inclusions, is given for all male (*n* = 5) (**G**) and female (*n* = 5) (**H**) exposure groups. Scale bars correspond to 400 μm (**A**,**D**) and 30 μm (**B**,**C**,**E**,**F**). * indicates significant difference to the control (*p* < 0.05).

For all control (no treatment) and vehicle control rats ((Hanks Balanced Salt Solution (HBSS) only)) there was a normal appearance and cellularity in all brain areas, including the hippocampus, dentate gyrus (Figure 1A,B), striatum, substantia nigra and prefrontal cortex (Figure 2A–H). There was also normal microgial immunoreactivity, and no specific positive staining for alpha-synuclein, hyper-phosphorylated tau, β-amyloid or pathological TDP-43 in any brain regions of control and vehicle control rats (Figure 3, Figure 4, Figure 5, Figure 6, Figure 7 and Figure 8).

For rats exposed to BMAA on PND3 the largest observed reduction in neuronal density was observed in the CA1 and CA3 regions of the hippocampus, with most loss in the CA1 region (Figure 1D–F). Severe neuronal loss was also observed in the upper and lower blades of the dentate gyrus together with severe thinning of the dentate gyrus blades (Figure 1D) compared to that of control rats (Figure 1A). Especially the lower blade of the dentate gyrus had several shrunken, pyknotic neurons (Figure 1E). Quantitative neuronal counts hematoxylin and eosin (H&E), and confirmed using a cresyl violet Nissl stain, revealed that rats exposed to 400 mg/kg BMAA on PND3 had frank neuronal loss in the hippocampus that ranged from 52 to 56% (Figure 2A,B). Rats exposed to a single dose of BMAA on either G14, PND4, PND5, PND6, PND7 or PND10 also exhibited marked neuronal loss in the hippocampal formation and dentate gyrus, but less than that observed in PND3 exposed rats. The hippocampus, the brain region involved in spatial, semantic and episodic memory (reviewed by [56]), is particularly vulnerable to neuronal reduction and typically exhibits neuronal dysfunction and neuropathological abnormalities in the earliest stages of AD and PD [57,58]. Furthermore, in advanced AD, the hippocampus is one of the most profoundly and consistently affected regions of the brain [59]. Interestingly, post-mortem examination of AD patients revealed that the neuronal populations most severely affected by AD are that of the CA1 (35% neuronal loss) and CA3 regions of the (20% neuronal loss) hippocampal formation [60,61,62] and that loss of neuronal density in the CA1 region strongly correlated with disease duration [63]. We thus present data here that demonstrate that a single neonatal BMAA exposure to rats on PND3, 4 and 5 produces a pattern of hippocampal neuronal loss similar to that usually observed in AD patients. Furthermore, the greater reduction in neuronal density of the hippocampus of male rats compared to female rats that was consistently observed for all exposure groups, also corresponds to that typically seen in AD patients with the CA1 region of male patients being markedly more damaged than that of females [64,65]. Furthermore, Regensburger et al. [66] have reported impaired adult neurogenesis of the hippocampus and subventricular zone in PD patients, and Camicioli et al. [67] reported progressive hippocampal volume loss in both AD and PD patients. Since the distribution of hippocampal neuronal loss in BMAA-exposed rats is similar to that observed in PD patients, it is plausible that BMAA could also exert its effect by either causing death and therefore progressive loss of hippocampal neurons, or alternatively, by impairing neurogenesis. The marked difference in vulnerability to BMAA toxicity when exposed on PND3, 4 and 5 when hippocampal neurogenesis peaks compared to exposure on G14, PND6, 7 and 10 (before and after the critical period of neurogenesis) suggests the latter. Interestingly, Kikisui and Mori [68], Lajud and Torner [69] and Loi et al. [70] have shown that a stressful stimulus can differentially alter the rate of neurogenesis in male and female neonatal rats. It is thus not unlikely that the gender-dependent differences in the extent of neuronal loss observed in this study could similarly be attributed to BMAA having a differential effect on male and female neurogenesis. Loss of TH-positive dopaminergic neurons in the substantia nigra *pars compacta* was limited to rats exposed to BMAA on PND3 and PND4, with BMAA being four times more damaging when administered on PND3 compared to PND4 (Figure 2E,F). Interestingly, no neuronal loss was observed in the ventral tegmental area (VTA), a region just adjacent to the substantia nigra *pars compacta*. Several studies have, however, shown that the VTA is much less affected in PD [71]. Neurons of the VTA are also much less vulnerable to other PD-assoicated toxins, such as 6-OHDA [72], MPTP [73], rotenone [74] and paraquat [75], than the substantia nigra *pars compacta*. These varying degrees of vulnerability between the VTA and substantia nigra *pars compacta* neurons have been attributed to differences in the expression of calbindin, a calcium-binding protein, and differences in expression levels of dopamine receptors [76] and the dopamine reuptake transporter (DAT) [77], but the underlying mechanism remains to be determined.

Severe neuronal loss was also observed in the dorsal striatum (caudate nucleus and putamen) of all postnatally, but not prenatally, BMAA-exposed rats. The extent of neuronal loss in the dorsal striatum was similar to the age-dependent neurodegeneration observed in the hippocampus. The most substantial neuronal loss in the dorsal striatum was observed in rats exposed to BMAA on PND3 (about 50% neuronal reduction) with markedly less reduction for each exposure day up to PND10. In PD, the neurodegeneration in this brain region usually starts in the dorsal striatum and extends to more ventral parts of the striatum as the disease progresses. The damage to the ventral striatum is, however, always less prominent than the damage to the dorsal striatum. Although the BMAA-exposed rats in this study only exhibited dorsal striatum pathology, these rats were relatively young at euthanisation (corresponding to approximately 20 human years) and, since AD and PD are commonly regarded as neurological diseases of old age, it is plausible that disease-symptoms would have markedly progressed as the rats aged. Yeterian and Pandya [78] and Hanganu et al. [79] proposed that, since the most rostrodorsal extent of the caudate head of the dorsal striatum is connected to the dorsolateral prefrontal cortex, damage or dysfunction of the caudate nucleus (i.e., as a function of dopamine depletion in PD and, potential alpha-synuclein overexpression as discussed below) would coincide with damage to the prefrontal cortex. Strikingly, in male rats exposed to BMAA on PND3, we observed a 10% neuronal loss in the prefrontal cortex and a marked, but lesser, reduction in neuronal density in female rats exposed at the same age. Degeneration of dopaminergic neurons in the substantia nigra *pars compacta*, as observed here, is typically considered to be the hallmark neuropathological feature of Parkinson’s disease [80], but multiple lines of evidence from anatomical and imaging studies indicate that cell loss or cell dysfunction in other brain regions, such as the hippocampus, striatum and prefrontal cortex, are also commonly seen in PD patients [81,82,83]. Neuronal loss in the hippocampus, prefrontal cortex and striatum is a contributing factor in the cognitive decline typically observed in PD and is proposed to be mediated, in part, by the decreased dopamine innervation into these brain regions after substantia nigra damage [84,85]. Dopaminergic neurons in the midbrain, particularly from the substantia nigra *pars compacta* and the ventral tegmental area innervate the hippocampal formation as well as the striatum [86]. Winner et al. [87] and Berg et al. [88] state that the neurotransmitter dopamine plays a pivotal role in hippocampal neurogenesis since dopaminergic fibers directly target hippocampal neural precursor cells. Interestingly, Ernst et al. [89] demonstrated that striatal neurogenesis also occurs well into adulthood and is also potentially modulated by dopamine. The loss of dopaminergic neurons in the substantia nigra *pars compacta* of BMAA-exposed rats would naturally result in a reduction of dopamine in the areas innervated by dopamine [90]. It is thus plausible that BMAA caused reduced neuronal densities in the hippocampus and striatum as a function of BMAA damaging the dopaminergic neurons in the substantia nigra *pars compacta* responsible for the recycling and release of dopamine for neurogenesis. The mechanism(s) by which BMAA could potentially cause damage to dopaminergic neurons of the substantia nigra *pars compacta* and hippocampal neurons remain, however, speculative. Interestingly, administration of 3,4-methylenedioxymethamphetamine (MDMA), a monoamine reuptake inhibitor and VMAT inhibitor [91,92] causes a pattern of neuronal loss in rats similar to what we observed following BMAA-exposure on PND3. MDMA-induced neurotoxicity is typically observed in the striatum and substantia nigra *pars compacta* [93], the hippocampus and the prefrontal cortex [94] of rodents. Administration of methamphetamine, a drug that elicits its effect by triggering a massive release of dopamine by displacing vesicles, by the inhibition of monoamine oxidase, and by enhancing the DAT-mediated reverse transport of DA transport across the plasma membrane [95] also typically results in neurodegeneration of TH-positive neurons in the substantia nigra and neuronal loss in the hippocampus, striatum and prefrontal cortex [96,97]. Scott and Downing [55] reported that rats exposed to BMAA on PND3, 4 and 5 demonstrated clinical symptoms and behavioural abnormalities that correspond to those observed in rats exposed to MDMA and methamphetamine, and subsequently proposed that BMAA might interfere with dopamine signaling. Interestingly, BMAA has been shown to cause alterations in serotonin and/or dopamine levels in the CNS of rats exposed neonatally [20] and in adulthood [98]. It is therefore not unlikely that BMAA potentially exerts its effect by interfering with dopamine and/or serotonin systems using similar modes as MDMA and/or methamphetamine. 

Our neuropathological data published here thus support and add to the data from the behavioural and cognitive studies published by Scott and Downing [55] that showed that rats are particularly susceptible to BMAA toxicity when exposed on PND3, presumably by altering dopamine and/or serotonin signaling in the developing brain and, subsequently also the adult central nervous system. Herlenius and Lagercrantz [99] demonstrated that the disruption of the normal timing or intensity of neurotransmitter signaling during the critical phases of brain development can lead to permanent changes in proliferation, differentiation and growth of their target cells and suggested this to provide the underlying mechanism for neurological abnormalities in adulthood.

We examined the presence of β-amyloid pathology and abundance (plaque deposition as well as β-amyloid build up in cerebral blood vessels) in the brain tissue of rats exposed to BMAA on G14, PND3, 4, 5, 6, 7 and 10 (Figure 3). Male rats exposed to BMAA up to PND3, 4 and 5, and female rats exposed to BMAA on G 14 and postnatally up to PND6 had moderate to severe levels of congophilic β-amyloid plaques, confirmed to be positive for β-amyloid using IHC, in the hippocampus and striatum. Interestingly, β-amyloid deposition was also observed in the prefrontal cortex of female rats exposed to BMAA on PND3 (Figure 3C). The spread of β-amyloid from the hippocampus to the prefrontal cortex, corresponding to the observed distribution of neuronal loss, in BMAA-exposed female rats and not in male rats suggests that BMAA-induced neuropathology might progress at a different rate in female BMAA-exposed rats compared to male rats, or, alternatively, that the prefrontal cortex of the female rat is more vulnerable to BMAA toxicity. A long-term comparative study that examines the progression of observed AD and PD-like symptoms and histopathology in male and female BMAA-exposed rats is required to address this possibility. Quantitative histological analysis of BMAA-exposed and control rats revealed the highest β-amyloid deposition, as high as 12% in males and 10% in females, in the hippocampus of rats exposed to BMAA on PND3. This is the brain area classically associated with AD-type pathology in humans [100]. A similar pattern of extracellular β-amyloid deposition was observed in the striatum, a brain region that commonly succumbs to amyloid plaques in AD and Down’s syndrome [101]. Male rats exposed to BMAA on PND3 and 4 as well as female rats exposed to BMAA on G14, PND3 and 4 had high β-amyloid burdens in the striatum, but less than that observed in the hippocampus for the same rat. The total amyloid β burden in the hippocampus and striatum was always higher for male BMAA-exposed rats compared to female rats that received the same treatment. Gershoni-Baruch et al. [102] have reported that amyloid deposition typically occurs at a lesser extent in females than in males, which Carrion et al. [103] attributed to a lower rate of amyloidogenesis due to suppression of amyloid formation by estrogen and progesterone in females. However, the presence of β-amyloid in the brain tissue of female rats exposed to BMAA on G14 and PND6 and 7, and not in male rats, could suggest that female rats have a longer window of susceptibility to BMAA toxicity (Figure 3J–M). This could potentially be attributed to differences in brain development such as the observed differences in neurogenesis observed between male and female neonatal rats [68], but this remains speculative. Male and female rats exposed to BMAA on PND3 and 5 exhibited cerebral amyloid angiopathy-like pathology (Figure 3G–I) with concomitant microbleeds and surrounding vasogenic oedema (Figure 4D,E) in the striatum. No edema or microbleeds were observed in any control animal sections. Microbleeds, as observed in 86% of AD patients [104], are caused by the deposition of Aβ in cerebral vessels that leads to weakening of artery walls and thus an increased risk of vessel rupture that could subsequently cause blood-breakdown products to leak into brain tissues adjacent from the damaged fragile vessels [105]. Interestingly, Chai et al. [106] reported increased cerebral microbleeds in the striatum of patients with chronic renal failure to be a significant risk factor of neurocognitive impairment. This suggests that the observed microbleeds in BMAA-exposed rats could contribute to the cognitive impairments in rats exposed to BMAA on PND3, 4 and 5 that was observed by Scott and Downing [55]. 

In the hippocampus of male rats exposed to 400 mg/kg BMAA on PND3, 4 and 5 and female rats exposed to BMAA on PND3, 4, 5, 6 and 10, B-amyloid plaques were often accompanied by intracellular hyper-phosphorylated tau positive NFTs (Figure 5A–C). NFT pathology was observed in female rats exposed to BMAA postnatally up to PND10, but in male rats only those exposed to BMAA on PND3, 4 and 5. Female neonatal rats thus seem to be susceptible to BMAA toxicity for a longer period during development than males. Quantitative histological examination using the AT8 antibody revealed that for PND3, 4 and 5 exposed rats, the males typically exhibited a greater NFT burden than females (Figure 5D,E) with the greatest number of inclusions observed in the CA1 region of the hippocampus. NFT formation in the CA1 region of the hippocampus is one of the earlier events in the pathogenesis of AD from where NFTs will spread, almost prion-like, to the amygdala, thalamus and isocortical areas [107]. We suspect that, as for neuronal loss, if rats exposed to BMAA neonatally were maintained for a longer term post-exposure, and thus euthanised in “old age”, more widespread tau-pathology would be observed. Importantly, although some of the other AD models such as rotenone and MPTP do manifest some hyper-phosphorylated tau deposition [108,109], these aggregates represent ‘pre-tangles’ and do not progress to frank NFTs [108,110,111] making this neonatal BMAA model the only non-transgenic rodent model available that exhibits this key neuropathology of AD. Arif et al. [112] reported that BMAA causes tau hyper-phosphorylation in hippocampal primary neurons, metabolically active brain slices and in vivo (i.c.v.infusion) potentially by inhibiting PP2A activity which is responsible for dephosphorylating most of the hyper-phosphorylated sites of tau [113] and is often compromised in the AD brain [114].

We also evaluated neuroinflammation in BMAA-exposed rats by immunohistochemical staining for activated microglia. All male and female rats exposed to BMAA on PND3, 4 and 5 (Figure 7C,D) had ongoing inflammatory processes, indicated by the presence of reactive microglial cells that were localised in the hippocampus (Figure 7A,B). In transgenic animal models of AD as well as human cases of AD, reactive microglia are always associated with dense-core plaques and diffuse β-amyloid deposits [115]. Meyer-Luehmann et al. [116] reported that microglial cells are typically recruited to plaques within 1–2 days of their appearance, but while microglial cells possess the ability to take up fibrillar forms of β-amyloid it seems as though the microglia have a difficult time degrading these dense aggregates in the AD brain and as a result do not effectively clear amyloid deposits in vivo [117]. In rats exposed to BMAA on PND3, 4 and 5, glial cells often contained deposits that stained positive with Thioflavin S near Thioflavin S-stained neurons, confirming that microglial cells are phagocytosing degenerating ThioS+ neurons in the hippocampus and striatum. Since Thioflavin S is a dye that only fluoresce when it binds to the paired helical tau filaments [118] and β-pleated sheet conformation of amyloid deposits [119], these phagocytosed neurons were presumably positive for intracellular tau-pathology. Microglial cells can typically degrade phagocytosed tau within 72 h, and the presence of ThioS+ deposits in microglial cells thus indicate that inflammatory responses and removal of degenerating neurons were still ongoing up to 72 h prior to euthanisation. Whether microglia are neuroprotective in the AD brain or facilitate and contribute to the neurotoxicity is currently still under active investigation, but several studies [120,121] have shown that the cognitive decline in AD is significantly correlated to the number of activated microglia rather than amyloid deposition in the diseased brain. Additionally, animal models have shown that the over-activation of microglia and the resulting continuous production of neurotoxic factors can initiate and amplify neuronal damage [122]. Two other in vivo BMAA studies have reported microgliosis in affected CNS regions and this is thus not unique to this exposure regime. Karlsson et al. [32] showed a moderate increase in microglial activation, but without accompanying β-amyloid plaques or NFTs, in the CA1 region of the hippocampus six months after BMAA exposure on PND9-10, and Yin et al. [35] reported microglial infiltration and some microglial nodules surrounding degenerating motor neurons of the spinal cord of adult rats exposed to BMAA via intrathecal infusion. 

One of the more remarkable findings in this study was the presence of alpha-synuclein positive Lewy bodies, the pathological hallmark of PD, in several remaining neurons of the substantia nigra *pars compacta* of male rats exposed to 400 mg/kg BMAA on PND3, 4 and 5 and in female rats exposed to BMAA on PND3, 4, 5 and 6 (Figure 7C,D), and not in the brain tissue of control and vehicle control rats (Figure 7A,B). In several of the widely used rodent PD models, such as 6-OHDA, neuronal loss in the substantia nigra *pars compacta* is not accompanied by the formation of Lewy body inclusions normally found in PD patients (reviewed by [123]). Only paraquat and rotenone treatment have been shown to increase alpha-synuclein expression in rodents [124]. This therefore makes this one of the few non-transgenic rodent models, and the only BMAA animal model, with the full spectrum of neuropathology typically seen in PD patients. Data from quantitative histology indicated that, similar to what was observed for the other examined proteinopathies, male BMAA-exposed rats had a greater alpha-synuclein burden in the substantia nigra *pars compacta* than females, with males exposed to BMAA on PND3 having an average of 8.38% dopaminergic neurons in the substantia nigra *pars compacta* harbouring Lewy bodies compared to 6.92% in female rats exposed on PND3 (Figure 7E,F). The exact mechanism for formation as well as the role, particularly whether it is neuroprotective or damaging, of Lewy bodies in dopaminergic neurons of the substantia nigra are still under debate (reviewed in [125]). However, alpha-synuclein has been shown to regulate the production of dopamine in cultured cells through its interaction with tyrosine hydroxylase, the rate-limiting enzyme responsible for converting tyrosine to l-3,4-dihydroxyphenylalanine (l-DOPA) in the dopamine synthesis pathway. Overexpression of alpha-synuclein in cell culture significantly reduced the activity of the tyrosine hydroxylase promoter and tyrosine hydroxylase activity, and wild-type alpha-synuclein markedly decreased striatal dopamine in several rodent models [126,127]. Additionally, Yavich et al. [128] showed that overexpression of alpha-synuclein decreased the rate of dopamine release both in mouse and cell culture models, but also showed that it was not attributable to changes in dopamine levels or clearance/uptake mediated by DAT. Nemani et al. [129] subsequently proposed that overexpression of alpha-synuclein affects the re-clustering of synaptic vesicles following endocytosis which causes a reduction in the size of the synaptic vesicle recycling pool and subsequently in an overall decrease in dopamine release. This could explain the direct proportional relationship between the extent of neuronal loss in the striatum and the total alpha-synuclein burden in the substantia nigra *pars compacta* of rats exposed to BMAA with striatal neuronal reduction of 18–48% observed in rats harbouring Lewy bodies as opposed to only a 0–6.8% reduction in striatal neurons in rats without Lewy bodies. Fujishiro et al. [130] additionally reported that the frequency of Lewy body inclusions in the substantia nigra *pars compacta* is typically proportional to the observed extent of cognitive impairment in PD and Lewy Body Dementia patients, and they subsequently suggested that dysfunction of the hippocampus, also as a function of Lewy body inclusions in the substantia nigra, could play a role in observed deficits. The severe neuronal loss and Lewy body inclusions in the substantia nigra *pars compacta* together with clinical symptoms, such as hindleg splay, whole body tremors and a low body posture, in rats exposed to BMAA on PND3 [55], make this a promising model for studying PD. 

In all male rats exposed to BMAA on PND3, 4 and 5 and in all female rats exposed to BMAA on PND3, 4, 5, 6 and 10, neuronal loss of the hippocampal formation was coupled to the presence of abundant cytoplasmic aggregates that stain positive for hyper-phosphorylated TDP-43 protein (Figure 8). In BMAA-exposed rats, prominent round and/or skein-like and/or diffuse pathological TDP-43 neuronal cytoplasmic inclusions could be observed in some, but not all, remaining neurons of the CA1, CA3 and CA4 regions of the affected hippocampus, the dentate gyrus and the basal ganglia, with selective localization in the dorsal striatum (Figure 8D–F). The hippocampal pathological TDP-43 load correlated well with the level of hippocampal sclerosis observed in the test brain samples. Presence of pathological TDP-43 positive aggregates is typically considered to be the hallmark pathological feature of ALS. However, the association of hyperphosphorylated TDP-43 aggregation with hippocampal sclerosis, defined as severe neuronal loss coupled to microgliosis in the CA1 region (reviewed in [131]), as observed in rats neonatally exposed to BMA on particularly PND3, 4 and 5 in the current study, is commonly associated with age-related pathologies such as dementia and Alzheimer’s Disease [132]. Interestingly, abnormal TDP-43 immunopositive neuronal cytoplasmic inclusions in the brain tissue are seen in 40–50% of AD cases, especially in those with more severe clinical manifestations of the disease [131]. 

It is important to note that the accumulative dose of G14, PND5 and PND10, did not produce a significantly greater neuronal loss and/or proteinopathy burden than for rats only exposed to BMAA on PND5 (data not shown), indicating the importance of BMAA exposure age over total perinatal dose.

### 2.3. Spinal Cord Pathology

**Figure 9:**
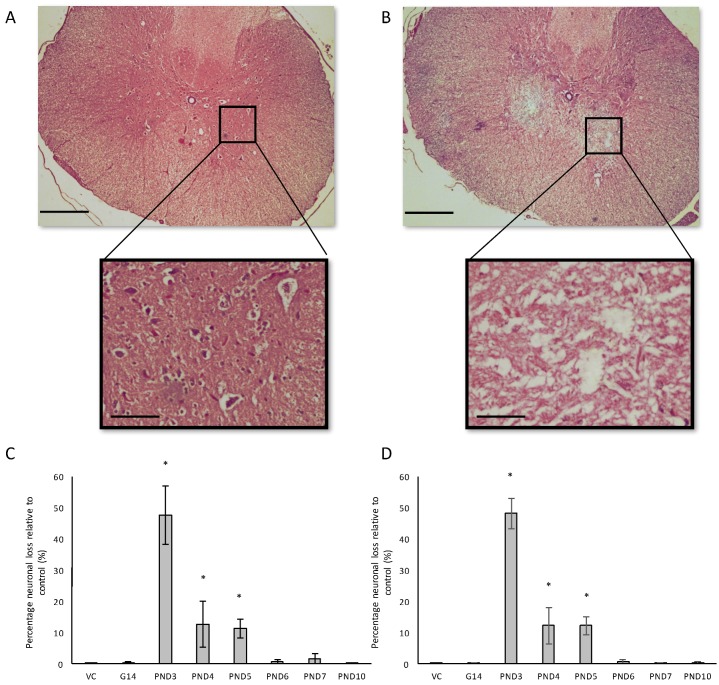
Comparison of motor neuron loss in the ventral horn of a 120-day old, PND5 BMAA-exposed male (**A**) compared to a control animal of the same age (**B**). The percentage neuronal loss in the ventral horn of the spinal cord, quantified by stereological counting of H&E and cresyl violet Nissl stained sections, for male (*n* = 5) (C) and female (*n* = 5) (**D**) rats are given for all exposure groups. * indicates significant difference to the control (*p* < 0.05). Scale bars correspond to 500 μm (**A**,**B**) and 50 μm for magnified images.

**Figure 10:**
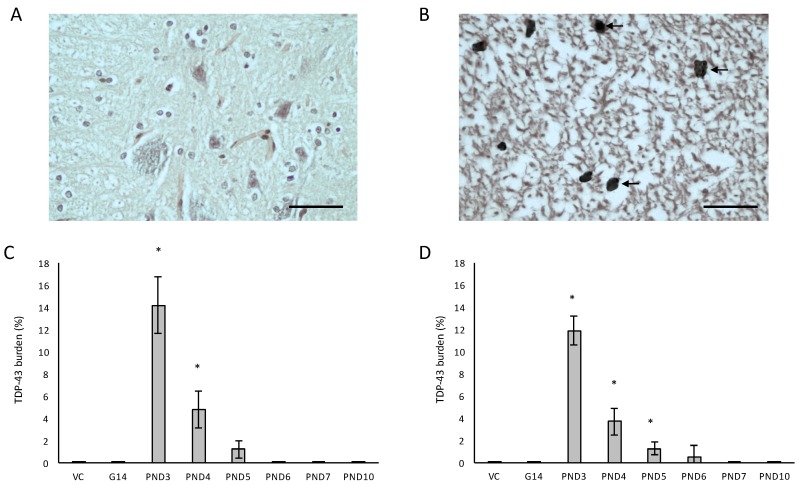
Neurons with pathological TDP-43 inclusions (indicated by arrowheads) in the few remaining neurons of the ventral horn of the spinal cord of a 120-day old male rat exposed to 400 mg/kg on PND3 (**B**) compared to the lack of pathological TDP-and overall normal appearance of the spinal cord of a male rat exposed to the vehicle control on PND3 (**A**). The percentage pathological TDP-43 burden, defined as the percentage neurons in the ventral horn of the spinal cord that harbor TDP-43 positive inclusions, is given for all male (*n* = 5) (**C**) and female (*n* = 5) (**D**) exposure groups. * indicates significant difference to the control (*p* < 0.05). Scale bars correspond to 50 μm.

Severe neuronal loss was observed in the spinal ventral horn, and not the dorsal horn, of all BMAA-exposed groups (Figure 9B–D). Quantitative histology demonstrated that the extent of neuronal loss was age-dependent with rats exposed to BMAA on PND3 having the greatest observed reduction in cellular volume. Abundant pathological TDP-43 immunopositive cytoplasmic inclusions were also observed in remaining neurons of the ventral horn (Figure 10) without concomitant pathological FUS cytoplasmic aggregates. TDP-43 positive, but FUS negative, inclusions in the spinal cord are consistent with classical features of late-onset amyotrophic lateral sclerosis. Yin et al. [35] similarly observed significant ventral horn, and little dorsal horn, pathology following intrathecal BMAA exposure to adult rats. Interestingly, the ventral horn (referred to as the anterior horn in humans) is also exclusively affected in the spinal cords of ALS patients [133]. Fucillo et al. [134] showed that although the spinal ventral horn loses motor neurons over time, it is initially able to self-regenerate, a process shown to be influenced by various signals in the developing ventral spinal cord of zebrafish and other vertebrates [135]. Interestingly, McLean and Fetcho [136] showed that axons from diencephalic dopaminergic neurons invade the spinal cord during neuronal differentiation and provide the only source of dopamine in the spinal cord of zebrafish and hence suggested that dopaminergic projections from the brain to the spinal cord could be an excellent candidate of modulating regeneration in the spinal cord. Furthermore, dopamine has been identified as a factor influencing neurogenesis in the developing brain [137] and adult brain [138] and the same could potentially be true for the spinal cord. The observed reduction in dopaminergic neurons of the substantia nigra *pars compacta* of BMAA-exposed rats together with the presence of abundant Lewy bodies, known to negatively influence dopamine recycling and release, would typically result in reduced levels of dopamine being available to the rest of the CNS. It is therefore certainly plausible that reduced dopamine signaling could be involved in the degeneration, or lack of regeneration, observed in spinal cord motor neurons of rats exposed to BMAA. 

Remarkably, small TDP-43-positive inclusions were also observed in the hypoglossal nucleus in the cervical spinal cord of two male rats exposed to BMAA on PND3. Damage to the hypoglossal nerve, that innervates all the extrinsic and intrinsic muscles of the tongue, is commonly associated with dysphagia in ALS and PD patients [139] and Tan et al. [140] reported that the presence of TDP-43 in the hypoglossal nucleus can be used to accurately diagnose ALS, and particularly end-stage ALS [141]. 

All neuropathologies, irrespective of type and location, were significantly correlated with each other using the Pearson product moment correlation. The strongest correlations (greater than 0.95) were ventral spinal cord neuronal loss: TDP-43 burden in the spinal cord (*r* = 0.9582), and neuronal loss in the prefrontal cortex:amyloid burden in prefrontal cortex (*r* = 0.9582). These correlations between neuronal loss and proteinopathies in the same regions are to be expected. Remaining correlations greater than 0.9 were, in order of significance, between microgliosis and amyloid in the hippocampus (*r* = 0.9483), neuronal loss in the spinal cord and loss of dopaminergic neurons in the substantia nigra (*r* = 0.9481), microgliosis and hyperhosphorylated tau burden in the hippocampus (*r* = 0.9432), alpha-synuclein burden in the substantia nigra and neuronal loss in the substantia nigra (*r* = 0.9409), beta-amyloid in the striatum and striatal neuronal loss (*r* = 0.9387), amyloid in the hippocampus and striatal neuronal loss (*r* = 0.9385), beta-amyloid in the hippocampus and hippocampal neuronal loss (*r* = 0.9345), microgliosis and hippocampal neuronal loss (*r* = 0.9283), hyperphosphorylated tau burden in the hippocampus and neuronal loss in the hippocampus (*r* = 0.9281), TDP-43 in the spinal cord and loss of dopaminergic neurons in the substantia nigra (*r* = 0.9281), neuronal loss in the spinal cord and in the striatum (*r* = 0.9220), alpha synuclein burden in the substantia nigra and amyloid in the prefrontal cortex (*r* = 0.9194), amyloid in the striatum and neuronal loss in the ventral spinal cord (*r* = 0.9109), beta-amyloid in the prefrontal cortex and hippocampal neuronal loss (*r* = 0.9056). Correlations of all other burdens and pathologies were greater than 0.7 (see Table S1, Supplementary Materials). 

## 3. Conclusions

Although Karlsson et al. [22,32] were able to show some mild neuronal loss in the CA1 and CA3 regions of the hippocampal formation following BMAA exposure on PND9–10, this is the first BMAA animal model that reproduces the severe levels of neuronal loss (>50%) that corresponds to that typically observed in the hippocampus of advanced AD patients. Furthermore, this is also the first study that demonstrates a reduction in TH-positive dopaminergic neurons in the substantia nigra *pars compacta* following systemic BMAA administration to rats and the only BMAA rodent model that has shown cellular volume loss and proteinopathies in the hippocampus, striatum and prefrontal cortex following BMAA exposure. Additionally, this is the only BMAA animal model able to demonstrate alpha-synuclein positive Lewy body inclusions in the remaining neurons of the substantia nigra *pars compacta* and the first rodent model to reproduce frank NFTs as observed in AD. It is also the only BMAA model that has reproduced degeneration, or possibly a lack of regeneration, and pathological TDP-43 inclusions in the ventral horn of the spinal cord using a systemic mode of administration. This BMAA model therefore exhibits Aβ, NFT of hyper-phosphorylated tau, pathological TDP-43 inclusions and Lewy bodies together with accompanied neuronal loss in relevant regions in the brain and spinal cord tissue of exposed rats, making it the only model to exhibit all of these histopathologies associated with ALS/PDC. The accumulative dose of G14, PND5 and PND10, did not produce a significantly greater neuronal loss and/or proteinopathy burden than for rats exposed to BMAA on PND5 and thus indicates the importance of BMAA exposure age over total perinatal dose. Finally, data from this study clearly demonstrates age and gender-dependent neuropathological deficits following administration of 400 mg/kg BMAA to rats. We always report here that BMAA exposure on particularly PND3, but also PND4 and 5, the critical period of neurogenesis in the rodent brain, is substantially more toxic than exposure to BMAA on G14, PND6, 7 and 10. Furthermore, our neuropathological data published here support and add to the data from behavioural and cognitive studies observed by Scott and Downing [55] that indicated that neurobehavioural abnormalities were particularly pronounced in rats exposed to BMAA on PND3. We suggest that BMAA may cause behavioural and cognitive deficits and histopathology by altering dopamine and/or serotonin signaling in the developing brain, and subsequently also the adult central nervous system, potentially by mechanisms similar to that of MDMA, reserpine or methamphetamine. We further suggest based on these data that BMAA may well be the causative or primary contributory agent in the development of ALS/PDC as observed on Guam, due to early life stage exposure to this potent neurotoxin.

## 4. Materials and Methods

### 4.1. Chemicals

β-*N*-methylamino-l-alanine (BMAA) was purchased from The Brain Chemistry Labs, Wyoming, USA and the purity confirmed as described by Banack et al. [142]. 

### 4.2. Animal Maintenance

Pregnant outbred Sprague Dawley rats were obtained from North-West University Animal Facility (South Africa) when they were on gestational (G) day 12. The dams were housed alone in polypropylene cages with wired mesh lids containing wood shaving bedding, nesting material and enrichment items. The litters were cross-fostered at PND3. Pups were randomly assigned to groups of minimum 10 pups per litter, with a homogeneous distribution of males and females as far as possible. The litter weights were monitored on PND1, 4, 7, 9, 14, 19, and 22 and after that every two weeks. After weaning on PND23 and onwards, five gender-matched rats were housed together in their respective treatments groups in standard polypropylene cages that contained wood shaving bedding and enrichment items. After weaning, the original dams were excluded from the experiment. The animals were housed in a temperature and humidity controlled environment with a 12 h light cycle beginning at 05:30 and maintained on a standard rodent diet (Epol^®^ specialised rodent cubes) that was provided *ad libitum*, as was water. Animals were randomised into exposure groups and subsequently identified by numerical markings on the tails. All animal experiments were approved by the Nelson Mandela Metropolitan University Research Ethics Committee—Animal (Project reference number A15-SCI-BCM-001, Date of Approval: 23 May 2015) and were conducted in accordance with national and institutional guidelines for the protection of animal welfare. 

### 4.3. Exposure

β-*N*-methylamino-l-alanine (BMAA) was dissolved in standard Hanks Balanced Salt Solution (HBSS) and a single dose of 400 mg/kg injected subcutaneously in a volume of 2 mL/kg body weight into both male and female Sprague Dawley pups on postnatal day 3, 4, 5, 6, 7 or 10. Vehicle control animals received equivalent injections of HBSS vehicle, which does not itself produce any toxicity. A 31-gauge, 8 mm needle attached to a 0.5-mL insulin syringe (BD Ultra-Fine^®^) was used to inject BMAA and/or vehicle into neonatal rats. Pregnant dams were injected with the same single dose (per weight of dam) on gestation day 14 to evaluate pre-natal exposure. Another exposure group was exposed to BMAA prenatally and again on postnatal days 5 and 10 to test the effect of an accumulative BMAA dose.

### 4.4. Histology and Immunohistochemistry

The animals were humanely euthanised at 120 days of age, the brain and spinal column dissected and immersion fixed in cold 10% phosphate-buffered formalin (pH 7.4) for histopathological examination. After fixation, the samples were embedded in paraffin and cut in 5-µm coronal sections at the levels of the olfactory bulb, frontal cortex, basal ganglia, striatum, thalamus, hippocampus, and mesencephalon, including substantia nigra and pons and routinely processed for histology and immunohistochemistry (IHC). All samples were blinded at the microtome. Brain and spinal cord sections from all animals were stained with hematoxylin and eosin (H&E), and relevant sections additionally stained for tyrosine hydroxylase (TH-positive), and examined by light microscopy. Consecutive sections were stained with Congo Red, for the identification of amyloid, and Thioflavin S with a thionine Nissl counterstain for the confirmation of amyloid plaques the and detection of neurofibrillary tangles. 

Serial paraffin sections from the same brain levels as examined for H&E, Congo Red and Thioflavin S staining were used for IHC with antibodies raised against amyloid-β (Aβ), tau protein, α-synuclein, tyrosine hydroxylase, hyperphosphorylated TDP-43 and reactive microglial cells. All immunostaining procedures, except that for tau protein, were conducted on the automated staining module Leica Bond-III. Sections were prepared using heat-induced epitope retrieval in citrate buffer, pH 6.0 and washed with phosphate buffer (pH 7.4) for 5 min (3 times). After washing, sections were blocked with 10% relevant normal serum with 3% Bovine Serum Albumin diluted in PBS for 30 min at room temperature, then incubated overnight with diluted primary antibody in a humidified chamber, to prevent tissue from drying out, at 4 °C. Immunostaining was performed for β-amyloid using an anti-amyloid β-A4 protein antibody (1:100 dilution; Merck Millipore, Cat. No. AB2500, Burlington, MA, USA) Reactivity was detected using the IHC-Select Detection Kit (Merck Millipore, Cat. No. DAB050, Billerica, MA, USA) and Mayer’s haemotoxylin was used as counterstain. Accumulation of phosphorylated paired helical filament tau (PHF-tau) was detected on the Leica Bond-Max automated staining system per Steffen et al. (2016) with an antibody against phosphorylated tau (1:50; anti-phospho-PHF-tau (AT8), Thermofisher, Cat No. MN1020, Waltham, MA, USA) and slides developed using Bond™ Polymer Refine Detection kit. Sections for alpha-synuclein analysis were incubated with sheep anti-aSyn antibody (1:500 dilution in 1% normal donkey serum in PBS containing 0.5% Triton X-100; Abcam, Cat. No. ab6162, Abcam, Cambridge, MA, USA), followed by washing with PBS containing 0.5% Triton X-100. The sections were then incubated with donkey anti-sheep HRP-conjugated secondary antibody for 2 h (1:500 dilution in 1% normal serum in PBS containing 0.5% Triton X-100; Abcam, Cat No. ab6900, Cambridge, MA, USA). The antigen–antibody complexes were identified following incubation with 0.05% 3,3′-diaminobenzidine (DAB) and 0.03% H_2_O_2_ solution. Sections containing the substantia nigra were additionally stained for tyrosine hydroxylase (TH) using rabbit polyclonal antibody against TH (1:750 dilution in 1% normal serum in PBS containing 0.5% Triton X-100; Abcam, Cat No. ab112) and subsequently incubated with biotinylated goat anti-rabbit IgG at 37 °C for 50 min, washed again as above, incubated with Horseradish peroxidase labelled streptavidin fluid at 37 °C for 30 min, washed, followed by DAB solutions for 5 min, washed and counterstained with Mayer’s hematoxylin for 3 min. For pathological TDP-43 detection sections were stained with TDP-43 rabbit monoclonal anti-hyperphosphorylated pathological TDP-43 antibody (1:3,000 dilution; Abcam), processed and mounted. Slides stained without primary antibody (only antibody diluent in the primary antibody step) served as negative controls. Relevant positive controls were run in parallel. Presence of NFT, β-amyloid deposits, alpha-synuclein positive inclusions and pathological TDP-43 positive inclusions were identified from blinded review by two independent neuropathologists (National Health Laboratories, South Africa and Stellenbosch University, South Africa). Abnormalities from immunostained tissue sections as well as H&E stained sections were first quantified per area using manual counting in eight sections in series from non-overlapping brain regions before confirmation using a Bioquant stereology semi-automated image analysis system (R&M Biometrics) with a random unbiased sampling scheme. All staining and image analysis procedures were performed by an individual blinded to the experimental study. Total β-amyloid burden (defined as the percentage of test area occupied by β-amyloid) and NFTs, alpha-synuclein and pathological TDP-43 burdens (defined as the percentage of neurons with the inclusion) were quantified for all relevant brain regions on coronal plane sections. 

### 4.5. Statistical Analysis

Due to the number of replicates in the study, no assumptions concerning equal sample variance or normal distribution of data were made, and the non-parametric Mann-Whitney *U* test (α = 0.05) was used to determine the statistical validity of data presented here.

## 5. Patents

Downing, T.G. (2017) Animal Model of Neurodegenerative Disease. United Kingdom—Provisional Patent Application No. 1705535.1 in the name of Nelson Mandela Metropolitan University entitled; Filing date: 5 April 2017.

## Supplementary Materials

The following are available online at http://www.mdpi.com/2072-6651/10/1/22/s1, Figure S1: Pearson correlation coefficients (*r*) calculated for quantified burdens and observed neuronal loss for all affected brain regions of male and female rats from all exposure and control groups (*n* = 100).
