# Supplementary material for: A Single Neonatal Exposure to BMAA in a Rat Model Produces Neuropathology Consistent with Neurodegenerative Diseases
Source: Toxins (Basel). 2017 Dec 29;10(1):22. doi: 10.3390/toxins10010022 (PMC5793109; doi:10.3390/toxins10010022)
Supplement: Supplementary file 1 [file toxins-10-00022-s001.pdf]

Supplementary Materials: A single neonatal exposure to BMAA in a rat model produces neuropathology consistent with neurodegenerative diseases

Laura Louise Scott [1,*] and Timothy Grant Downing [2,*]

Table S1. Pearson correlation coefficients (*r*) calculated for quantified burdens and observed neuronal loss for all affected brain regions of male and female rats from all exposure and control groups (*n* = 100).

	PFC Loss	SN Loss	HC Loss	Striatum Loss	SC Loss	Amyloid: HC	Amyloid: Striatum	Amyloid: PFC	Tau: HC	a-syn	TDP-43: HC	TDP-43: SC
**SN Loss**	0,8336											
**HC Loss**	0,8171	0,7392										
**Striatum Loss**	0,6913	0,8784	0,8444									
**SC Loss**	0,8038	0,9481	0,7016	0,9220								
**Amyloid: HC**	0,7415	0,7879	0,9345	0,9285	0,8757							
**Amyloid: Striatum**	0,8668	0,8438	0,8896	0,9387	0,9109	0,8668						
**Amyloid: PFC**	0,9582	0,8731	0,9056	0,8925	0,8169	0,8977	0,8918					
**Tau: HC**	0,7905	0,8768	0,9281	0,8167	0,8330	0,8845	0,8239	0,7839				
**a-syn**	0,8533	0,9409	0,8448	0,8582	0,8462	0,8792	0,8594	0,9194	0,8928			
**TDP-43: HC**	0,8100	0,8222	0,8944	0,8293	0,7057	0,8930	0,8493	0,8394	0,8894	0,7794		
**TDP-43: SC**	0,8432	0,9281	0,8492	0,8139	0,9581	0,8790	0,9055	0,8103	0,7583	0,8933	0,8392	
**Microgliosis**	0,8732	0,8124	0,9283	0,8237	0,7083	0,9483	0,8429	0,9038	0,9432	0,8594	0,8943	0,8329

PFC: Prefrontal Cortex; SN: Substantia Nigra; HC: Hippocampus; SC: Spinal cord; Tau: Hyperphosphorylated Tau; a-syn: alpha-synuclein
